# *Trichodysplasia spinulosa*-Associated Polyomavirus (TSV) and Merkel Cell Polyomavirus: Correlation between Humoral and Cellular Immunity Stronger with TSV

**DOI:** 10.1371/journal.pone.0045773

**Published:** 2012-09-24

**Authors:** Arun Kumar, Anu Kantele, Tommi Järvinen, Tingting Chen, Heli Kavola, Mohammadreza Sadeghi, Klaus Hedman, Rauli Franssila

**Affiliations:** 1 Departments of Virology, Haartman Institute, University of Helsinki, Helsinki, Finland; 2 Department of Bacteriology and Immunology, Haartman Institute, University of Helsinki, Helsinki, Finland; 3 Department of Medicine, Institute of Clinical Medicine, University of Helsinki, Helsinki, Finland; 4 Division of Infectious Diseases, Helsinki University Central Hospital, Helsinki, Finland; 5 Department of Plastic Surgery, Helsinki University Hospital, Helsinki, Finland; 6 Helsinki University Central Hospital Laboratory Division, Helsinki, Finland; University of Cape Town, South Africa

## Abstract

Merkel Cell Polyomavirus (MCV) is a common infectious agent likely to be involved in the pathogenesis of most Merkel cell carcinomas (MCC). *Trichodysplasia spinulosa*-associated polyomavirus (TSV), which exhibit high seroprevalence in general population, has been detected in *trichodysplasia spinulosa* (TS) skin lesions suggesting an etiological role for this disease. Previous studies have shown strong MCV-specific T-cell responses, while no data exist on T-cell immunity against TSV. In order to characterize Th-cell immunity against TSV, and to allow comparisons with the MCV-specific Th-cell immunity, we studied TSV-specific proliferation, IFN-γ, IL-10 and IL-13, and MCV-specific IFN-γ and IL-10 responses in 51 healthy volunteers, and in one MCC patient. Recombinant TSV and MCV VP1 virus-like particles (VLPs) were used as antigens. A significant correlation was found between virus-specific Th-cell and antibody responses with TSV; with MCV it proved weaker. Despite significant homology in amino acid sequences, Th-cell crossreactivity was not evident between these viruses. Some subjects seronegative to both TSV and MCV exhibited Th-cell responses to both viruses. The agent initially priming these Th-cells remains an enigma. As CD8**^+^** cells specific to MCV T-Ag oncoprotein clearly provide an important defense against established MCC, the MCV VP1-specific Th-cells may, by suppressing MCV replication with antiviral cytokines such as IFN-γ, significantly contribute to preventing the full process of oncogenesis.

## Introduction

Polyomaviruses are small, non-enveloped double stranded DNA viruses. In 2008, Feng et al. discovered a new polyomavirus, Merkel cell polyomavirus (MCV) accounting for most cases of Merkel cell carcinoma (MCC), an aggressive neuroendrocrine cancer of the skin [1], [2]. Clonally integrated into the MCC genome, the viral sequences have been shown to be present in 24–89% of MCC tumors worldwide, [3]–[8]. HPyV6 and HPyV7, two human polyomaviruses newly discovered, also seem to have a predilection for the skin. Serological studies have demonstrated infections by these three dermatotropic polyomaviruses to be very common [9]–[13].

A new polyomavirus, TSV, was recently identified in an immunosuppressed individual with this rare, severe skin disease, *trichodysplasia spinulosa* (TS) [14]. This disease is characterized by spicules, i.e. follicular papules and keratin spines that become widespread on the face, sometimes accompanied by alopecia of the eyebrows and lashes, and, in some cases, leading to facial distortion. Histopathology of the affected skin showed distended and abnormally matured hair follicles with high numbers of inner root sheath cells containing excessive amounts of trichohyalin features. Electron microscopic studies revealed the presence of polyomavirus-like particles in skin biopsies of TS patients, which accords with the etiological role of TSV for this disease [15]–[20]. Even if infections by TSV occur frequently, they appear to become symptomatic only in immunocompromised patients. The existing data indicate that TSV infections are frequent among the general population (∼ 70% seroprevalance), and that primary infection often occurs in childhood [21], [22].

CD4^+^ cells play important roles in antiviral immunity by producing antiviral cytokines, providing help for B cells in antibody production, as well as generating cytotoxic and memory CD8^+^ T-cell populations. Recent studies have defined additional functions for CD4^+^ T-cells in enhancing innate immune responses and mediating non-helper antiviral effector functions [23], [24]. We and a few others have recently shown T-cells to be important mediators of MCV-specific immune surveillance [25], [26].

In this study we compared the characteristics of Th-cell immunity with the two dermatotropic human polyomaviruses, MCV and TSV. We investigated proliferation, IFN-γ IL-10, and IL-13 cytokine responses by stimulating PBMC with MCV or TSV VP1 virus-like particles (VLP). IFN-γ is an archetype II interferon [27] which has direct antiviral activity, enhancing cellular cytotoxicity, and acting as a critical extrinsic tumor-suppressor factor in immunocompetent hosts via several types of antitumor actions. While Th1-cells predominantly produce IFN-γ, also cytotoxic T-cells and NK cells give rise to this proinflammatory cytokine [27]–[31]. A therapeutic effect of type I and II IFNs on MCV-positive MCC cell lines has been shown recently [32].

IL-10 has potent immunosuppressive effects and serves a substantial role in the regulation of immune responses. Its major sources are Th-cells and a subset of regulatory T-cells. IL-10 inhibits Th1 cells, NK cells and macrophages. These three cell types are required for optimal pathogen clearance, and they also contribute to tissue damage during infection. In consequence, IL-10 can both impede pathogen clearance and ameliorate immunopathology [33], [34]. Besides these functions IL-10 also induces B-cell growth and IgG secretion, and is essential for the maintenance of the human germinal centre B-cells *in vitro* [35].

IL-13 is an important cytokine produced mainly by Th2 cells [36]. It has several unique effector functions including the regulation of gastrointestinal parasite expulsion, intracellular parasitism, airway hyperresponsiveness, allergic inflammation, and the class switch to IgE and IgG4 [37], [38]. In many studies IL-13 is suggested to have a contradictory role in tumor immunity [39]–[41].

To characterize the Th-cell immunity against TSV, and to allow comparisons to MCV-specific Th-cell immunity, we studied MCV and TSV-specific proliferation, IFN-γ IL-10 and IL-13 responses. As TSV and MCV VP1 are known to have significant structural similarities [14], we wanted to investigate whether it is possible to distinguish one Th-cell response from another.

## Materials and Methods

### Study Groups

Altogether 51 randomly selected healthy asymptomatic subjects, with no underlying diseases were studied: 30 were seropositive (15 men, 15 women, aged 19–58 years) and 21 seronegative (5 men, 16 women, aged 20–58 years) for TSV and 24 (8 men, 16 women, aged 19–58 years) and 27 (12 men, 15 women, aged 20–58 years) for MCV, respectively. The study protocol followed the human experimentation guidelines of the US Department of Health and Human Services in the conduct of clinical research and was approved by the ethics committee of the Department of Medicine at Helsinki University Central Hospital. Written informed consent was obtained from all volunteers.

MCV and TSV-specific Th-cell responses were also studied in a 48-year old MCC patient. The patient was HIV negative, and had no immunosuppressive medication or other known immunodeficencies. The tumor biopsies of skin and blood samples were analyzed by quantitative PCRs for MCV [42] and TSV [14] DNAs and by cytokine assays. Clinical data of this patient are enclosed as supplement (Note S1).

### Antigens for Proliferation and Cytokine Assays

**Figure 1:**
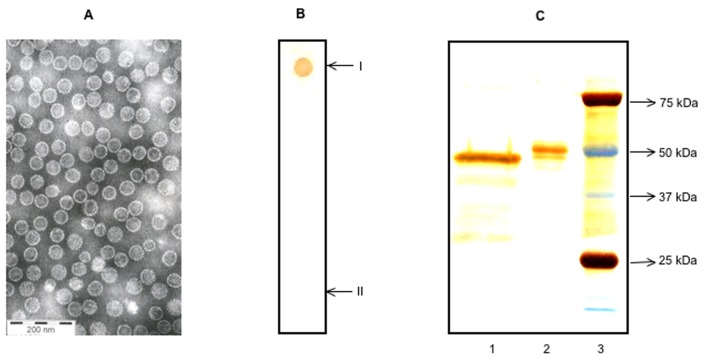
Characterization of antigens. EM of sterile TSV particles (A) purified by caesium chloride density gradient ultracentrifugation, with 200 nm scale bar shown. Dot blotting (B) for TSV antigen, studied with TSV-IgG positive (I) and negative (II) sera. Silver staining of capsid proteins (C) in 10% SDS PAGE. Lane 1: TSV VP1 capsid antigen, lane 2: MCV VP1 capsid antigen, lane 3: molecular weight markers.

The major capsid protein, virus protein (VP) 1 of MCV and TSV were expressed with recombinant baculoviruses in Sf9 cells and purified by CsCl gradient ultracentrifugation as described [22], [43]. After extensive dialysis the protein was concentrated and purified further with 50 KDa MWCO centrifugal filters (Amicon Ultra, Millipore, Billerica, MA). The purities of antigens were further characterized by silver staining (SilverXpress, Invitrogen, Carlsbad, CA, USA) using same amounts of antigens, and by dot blotting with seropositive human sera as described earlier [22], [25], [43]. EM with negative staining VP1 of TSV showed virus-like particles (Fig. 1). As a control antigen, we used in-house prepared and heat inactivated *Candida albicans*. Endotoxin in the antigen preparations was measured by the Limulus amebocyte lysate assay (QCL-1000; Cambrex Biosciences, Walkersville, MD, USA), and was less than 2 EU/mg with all the VLPs. Preparation of the working stocks and analyses of MCV and TSV antigens were carried out simultaneously.

### Antibody Assays

We measured TSV and MCV IgG by enzyme immune assay (EIA), employing as antigen virus-like particles as described previously in detail [22], [43]. For TSV, absorbance value >0.240 was determined as cutoff for being TSV IgG positive, whereas all those with values <0.100 were considered TSV IgG negative. One of 51 samples showed an absorbance value between these cutoffs. For this sample the TSV -VLP competition assay [22] was performed, after which this sample was regarded as seronegative.

### Isolation of PBMC

Blood was drawn to mononuclear cell separation tubes (Vacutainer CPT, Becton Dickinson, Franklin Lakes, NJ, USA). The tubes were centrifuged at 1500×g for 30 minutes and washed two times with PBS. PBMC were separated within 2 hrs of blood sampling [44], [45].

### Lymphocyte Culture

Isolated PBMC were resuspended in RPMI-1640 (Sigma, St Louis, MO, USA) containing 20mM HEPES, 2 mM L-glutamine, streptomycin (100 µg/ml), penicillin (100 U/ml), 50 µM 2-mercaptoethanol and 10% human AB serum (Cambrex Biosciences, USA) and cultured at 37°C; 5% CO_2_ [25], [44], [45]. MCV and TSV VLP were used at concentrations of 0.5 µg/ml and 1.5 µg/ml and *Candida albicans* control antigen at 2.5 µg/ml.

### Proliferation Assay

PBMC (200,000/well) were cultured with the antigens for six days (37°C; 5% CO_2_) in triplicate in 96 well U-bottom plates (Coster, Corning Inc., Corning, NY, USA) and pulsed for the last 16 hours with 1 µ Ci of tritiated thymidine (specific activity 50 Ci/mmol; Nycomed Amersham, Buckinghamshire, UK). Thymidine incorporation was measured in a scintillation counter (Microbeta, Wallac,Turku, Finland) [25], [44], [45]. The data were expressed as counts per minute (Δ cpm): Δ cpm  =  mean cpm (test antigen) – mean cpm (media).

### Cytokine Assays

PBMC culture supernatants were harvested after 3 days for IFN-γ and after 5 days for IL-10 and IL-13, and were stored at −20°C. Cytokine production in the supernatants was analysed by IFN-γ, IL-10 (Pharmingen, San Diego, CA, USA) and IL-13 (Invitrogen corporation CA, USA) kits, according to the manufacturers’ instructions. Background (media) cytokine production was subtracted from total to yield antigen specific cytokine production [25], [45]. The detection limits for IFN-γ, IL-10 and IL-13 were 5, 8 and 6 pg/ml, respectively.

### Depletion of CD4^+^ or CD8^+^ Cells

PBMC were depleted of CD4^+^ or CD8^+^ T cells by using magnetic beads coated with CD4- or CD8-specific monoclonal antibodies (Invitrogen Dynal AS, Oslo, Norway), according to the manufacturer’s instructions. Then, 200,000 pure CD4**^+^** or CD8**^+^** depleted cells were cultured with the antigens as described previously [25]. Efficiency of the depletion was analyzed by flow cytometry (Cyan, Beckman Coulter). The depleted PBMC were stained with RPE, FITC and APC (DakoCytomation, Glostrup, Denmark) labeled monoclonal antibodies for CD4, CD8 and CD3 T cells. CD3^+^ cells were 99% CD8^+^ and CD4^+^ after depletion of CD4 and CD8 T cells, respectively.

### Antibody Blocking Assays

Class restriction of the T-cell responses was further studied by HLA class II-specific MAbs (HLA-DR, DP, DQ) (IgG2a, clone Tu39; BD PharMingen), or isotype control MAb (IgG2a, clone G155–178; BD PharMingen). Briefly, PBMC were cultured with either HLA class II-specific or isotype control MAb and culture supernatants were collected on days 3 and 5 for cytokines analysis. These antibodies were used at 5 µg/ml, according to the manufacturer’s instructions.

### Statistical Methods

Responses among MCV and TSV seropositive and seronegative subjects were compared by using the Mann-Whitney U test. Paired responses were evaluated by using the Wilcoxon Signed Rank test. The correlations were studied with Spearman’s correlation. The distribution of responders against each antigen was studied using Fisher’s Exact test. P values <0.05 were considered significant. All analyses were done with a SPSS statistical program version 15.0.

## Results

### Comparison of TSV-specific Proliferation, IFN-γ, IL-10 and IL-13 Responses in the TSV-Seropositive and Seronegative Subjects

Table 1 shows the comparisons of the TSV-specific proliferation, IFN-γ, IL-10 and IL-13 responses among the 30 TSV-seropositive and 21 seronegative subjects. TSV-specific IL-10 responses were similar in these two groups, whereas proliferation, IFN-γ and IL-13 responses appeared to be stronger among the TSV-seropositive than -seronegative subjects. However, these differences did not prove statistically significant (Table 1). Background proliferation and cytokine responses were low and similar among TSV-seropositive and -seronegative subjects (Table 2).

**Table 1:** Comparison of TSV-specific proliferation and cytokine responses in 30 TSV seropositive and 21 seronegative subjects.

	Proliferation ΔCPM ±SD		IFN-γ pg/ml±SD		IL-10 pg/ml±SD		IL-13 pg/ml±SD
Ag (TSV VP1) concentration ( µg/ml)	1.5	0.5	1.5	0.5	1.5	0.5	1.5
**TSV seropositive**	*12092*±10796	*7858*±10538	128.2±163.8	126.4±214.6	56.2±92.8	25.3±51.9	*24.1*±29.1
**TSV seronegative**	*7112*±6508	*3434*±3583	75.3±92.3	50.8±76.9	52.2±65.5	21.2±32.4	*14.3*±20.1
**P**	0.095	0.145	0.301	0.362	0.878	0.653	0.184

**Figure 2:**
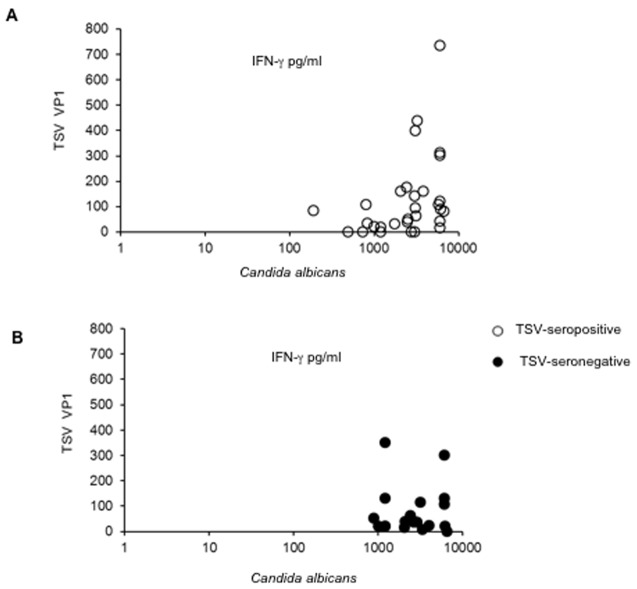
Correlation between TSV VP1 and *Candida albicans-*specific IFN-γ responses. Correlation analysis between TSV VP1 and *Candida albicans-*specific IFN-γ responses were analysed among TSV-seropositive (A) and seronegative (B) individuals.

Individual variation of TSV responses was extensive, causing large standard deviations in both groups, yet all subjects showed strong responses with the *Candida albicans* control antigen (Fig. 2).

**Table 2:** Comparison of background (Media) proliferation and cytokine responses among TSV-seropositive and seronegative subjects.

	Proliferation ΔCPM ±SD	IFN-γ pg/ml±SD	IL-10 pg/ml±SD	IL-13 pg/ml±SD
**TSV seropositive**	1047±794	3.4±7.2	7.6±4.4	1.3±3.6
**TSV seronegative**	1250±1468	4.3±6.0	9.0±4.8	0.3±1.7
**P**	**0.989**	**0.346**	**0.326**	**0.307**

### Comparison of MCV-specific IFN-γ and IL-10 Responses in the MCV-seropositive and Seronegative Subjects

In contrast to findings with TSV, the MCV-specific IFN-γ and IL-10 cytokine responses were significantly higher in the 24 MCV-seropositive than in the 27 MCV-seronegative subjects (P<0.0001) whereas the responses with *Candida albicans* control antigen were similar (P≥0.250) (Table S1).

### Identification of TSV-specific Cytokine Secreting Cells

**Figure 3:**
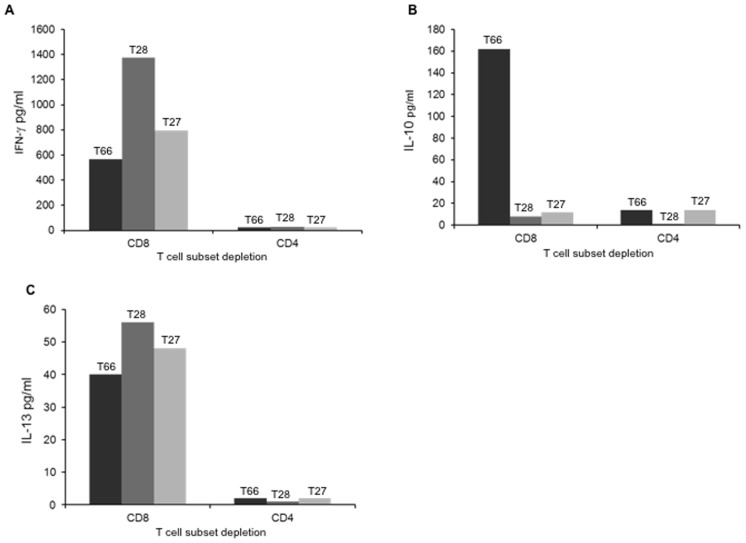
TSV VP1-specific T-cell responses after T-cell subset depletion. PBMC of two TSV seropositive (T66, T28) and one seronegative subject were deplete either of CD4**^+^** or CD8**^+^** T cells and stimulated with TSV VP1-VLPs (1.5µg/ml). IFN-γ (A), IL-10 (B) and IL-13 (C) were studied by ELISA.

PBMC were first depleted of either of CD4**^+^** or CD8**^+^** T cells by using monoclonal antibodies attached to magnetic beads. Altogether 3 subjects were studied, among them two were seropositive (T66, T28) and one was a seronegative “top responder” (T27) for TSV. TSV-specific IFN-γ L-10 and IL-13 secretion was readily detectable after depletion of CD8^+^ T cells, whereas the removal of CD4^+^ T cells strongly reduced the responses among all the subjects (Fig. 3).

**Figure 4:**
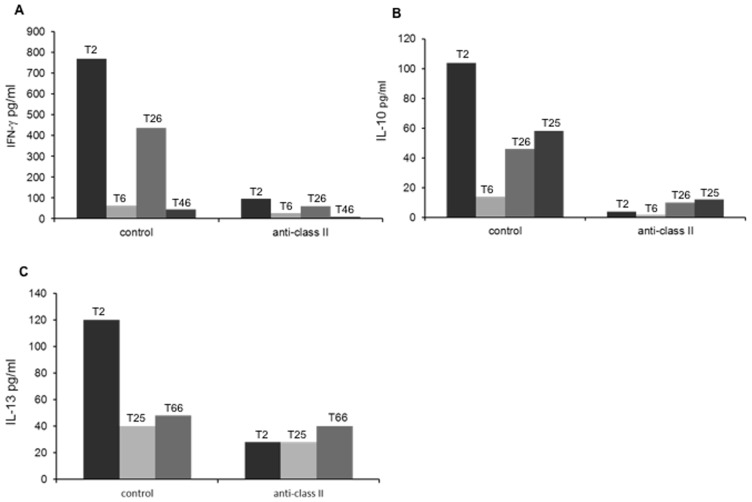
Effect of HLA class II-specific monoclonal antibodies on TSV-VP1 specific cytokine responses. PBMC of TSV seropositive individuals were incubated with HLA class II-specific monoclonal antibody and with a isotype-matched contol antibody (MAbs) and stimulated with TSV-VP1 for IFN-γ (A), IL-10 (B) and IL-13 (C) assessment.

Next, we studied the influence of class II-specific MAb (expected to prevent antigen presentation) and its isotype-matched control on Th-cell responses. TSV-specific cytokine responses were readily detectable with the isotype control MAb, whereas the HLA class II-specific MAb invariably reduced the responses (Fig. 4).

### The Effect of TSV-specific Humoral Response on TSV-specific Th-cell Responses

**Figure 5:**
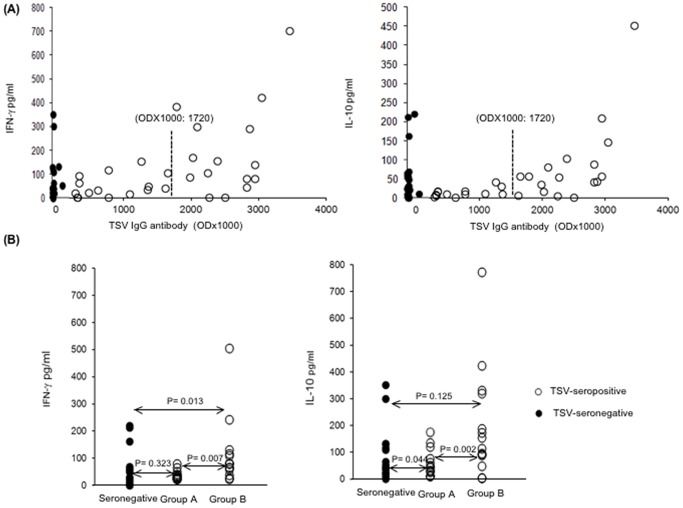
(A) Effect of TSV-specific IgG level on Th-cell responses. TSV-specific IFN-γ and IL-10 responses were plotted against TSV-IgG among seropositive (open circles) and seronegative individuals (closed circles). TSV VP1 specific humoral immune response is shown by optical density × 1000 (OD×1000). Median OD×1000 (1720) is shown by (–) a line. (**B**) TSV-specific IFN-γ and IL-10 responses were compared between the TSV- seronegative subjects and sub-groups of TSV-seropositive subjects. Group A: TSV-seropositive subjects having TSV-IgG OD×1000 below 1720, and group B: subjects having TSV-IgG OD×1000 above 1720.

First we studied whether there would be a correlation between the strength of TSV-specific humoral response, described as optical density × 1000 (OD×1000) and the Th-cell mediated responses (Fig. 5A). A significant positive correlation was found between humoral response and TSV-specific proliferation (P = 0.008), IFN-γ (P = 0.007) and IL-10 (P = 0.002) in the 30 TSV seropositive subjects; with IL-13 the correlation did not reach significance (P = 0.098). Data for IFN-γ and IL-10 are shown in Fig. 5A.

The effect of TSV-specific humoral responses on TSV-specific Th-cell responses was further studied by dividing the 30 TSV seropositive subjects into two groups of equal size (15 subjects) according to their TSV-IgG ODs (Fig 5B): subjects in group A had ODs under and those in group B had ODs above the median OD (1720) of the 30 TSV seropositive subjects. Group A had TSV-specific responses similar to that found in the seronegative controls. In group B, by contrast, both the TSV-specific proliferation (P = 0.007) and IFN-γ (P = 0.013) responses were stronger than in the seronegative controls (Fig. 5B); no difference was found in the TSV-specific IL-10 (P = 0.125) and IL-13 (P = 0.062) responses, or responses with the *Candida albicans* control antigen (P≥0.283).

Unanticipatedly, TSV specific proliferation (P = 0.008), as well as IFN-γ (P = 0.007) and IL-10 responses (P = 0.002) were all significantly stronger in group B than in group A. No significant difference was found in IL-13 responses (P = 0.098); proliferation and cytokine responses with the *Candida albicans* control antigen were similar (P≥0.251) in both groups. Data for IFN-γ and IL-10 are shown in Fig. 5B.

### The Effect of MCV-specific Humoral Response on MCV-specific Th-cell Responses

With MCV the correlations between humoral (MCV IgG ODx1000) and Th-cell responses were less evident among the 24 seropositive subjects, and the lowest P-value was 0.053 both with IFN-γ and IL-10 (Fig. S1A). MCV specific IFN-γ responses were higher among those with MCV-IgG levels above the median (874) than among those with values under this limit, both at 0.5 and at 1.5 µg/ml antigen concentrations, P = 0.020 and 0.028, respectively (Fig. S1A). These groups showed no differences in their MCV-specific IL-10 responses, P≥0.32 (Fig. S1A) or responses with the *Candida albicans* control antigen (P≥0.18) (data not shown).

MCV-seropositive subjects with ODx1000 under the median still showed significantly stronger IFN-γ responses than those found among the seronegative controls (P≤0.022) at both antigen concentrations, whereas with IL-10 the difference was significant (P = 0.049) only at MCV-antigen concentration of 0.5 and borderline (P = 0.056) at 1.5 µg/ml (Fig. S1B). No differences were found with the *Candida albicans* control antigen (Data not shown).

### The Effect of MCV-serostatus on TSV-specific Th-cell Responses

In order to study whether there is crossreactivity between MCV and TSV, the subjects were divided in four groups according to their serological responses (Table 3): MCV**^+^**TSV**^+^**, MCV**^−^**TSV**^+^**, MCV**^−^**TSV**^−^** and MCV**^+^**TSV**^−^**.

**Table 3:** Comparison of MCV and TSV-specific IFN-γ and IL-10 (pg/ml±SD) responses in the different groups (responses at 0.5 µ g/ml concentration are shown in parentheses below responses at 1.5 µ g/ml).

	Serostatus		IFN-γ			IL10		
Group	MCV	TSV	MCV	TSV	P	MCV	TSV	P
**1**	**Pos**	**Pos**	145.5±190.9	117.6±99.1	0.860	56.5±47.3	54.5±58.6	0.906
			*(114.0±150.9)*	*(101.7±140.3)*	*(0.701)*	*(33.1±25.3)*	*(23.0±18.2)*	(*0.147*)
**2**	**Neg**	**Pos**	43.2±58.0	136.3±202.8	**0.007**	21.8 ±18.8	57.6 ±114.3	0.270
			*(30.5±45.3)*	*(145.2±260.5*	*(0.086)*	*(11.6±15.8)*	*(27.1±69.0)*	*(0.687)*
**3**	**Neg**	**Neg**	44.6±48.9	76.3±106.2	0.860	40.3±49.8	56.6±85.5	0.670
			*(26.4±35.7)*	*(33.3±68.0)*	*(0.767)*	*(16.7±24.2)*	*(22.2±43.8)*	*(0.233)*
**4**	**Pos**	**Neg**	179.8±168.2	74.5±83.1	**0.003**	120.0 ±144.0	48.4 ±44.4	**0.004**
			*(189.8±173.2)*	*(66.8±84.1)*	***(0.003)***	*(56.0±31.9)*	*(20.3±21.5)*	***(0.005)***

Comparisons of TSV-specific responses between group MCV**^+^**TSV**^+^** and group MCV**^−^**TSV**^+^**, showed that MCV seropositivity caused no significant increases in TSV responses (P≥0.092). Likewise, comparisons between groups MCV**^−^**TSV**^−^** and MCV**^+^**TSV**^−^**, showed no significant differences (P≥0.132) (data not shown). Responses in groups MCV**^+^**TSV**^+^** and MCV**^+^**TSV**^−^** were also similar (P≥0.093), (data not shown), whereas significantly stronger TSV-specific proliferation, IFN-γ and IL-13 responses were detected in MCV**^+^**TSV**^+^** than in MCV**^−^**TSV**^−^** group (Table 4). TSV-specific IL-10 responses (Table 4) and those to *Candica albicans* control antigen were similar in both groups, (P≥0.18), (data not shown) respectively.

**Table 4:** Comparison of TSV-specific proliferation and cytokine responses among TSV ^+^ MCV ^+^ and TSV^–^ MCV^–^ groups.

	Proliferation ΔCPM ±SD		IFN-γ pg/ml±SD		IL-10 pg/ml±SD		IL-13 pg/ml±SD
TSV VP1 concentration ( µg/ml)	1.5	0.5	1.5	0.5	1.5	0.5	1.5
**TSV ^+^ MCV ^+^**	11996±7192	6026±4349	117.6±99.1	101.7±140.3	54.4±58.5	23.0±18.2	27.5±30.3
**TSV^–^ MCV^–^**	4574±4852	1456±1542	76.3±106.2	33.3±68.0	56.6±85.4	22.2±43.8	6.8±9.5
**P**	**0.025**	**0.008**	0.054	**0.025**	0.101	0.305	**0.015**

### The Effect of TSV-serostatus on MCV-specific Th-cell Responses

Strikingly, with the MCV antigen Th-cell responses were stronger even in the MCV**^+^**TSV**^−^** than in the MCV**^−^**TSV**^+^**control group. Responses with *Candida albicans* antigen were similar in these groups (P≥0.312) (Table S2).

### Comparison of MCV- and TSV-specific Th-cell Responses in Sub-groups

Th-cell cross-reactivity between TSV and MCV was further studied by comparing Th-cell mediated IFN-γ and IL-10 responses against TSV and MCV antigens *within* each of the groups MCV**^+^**TSV**^+^**, MCV**^−^**TSV**^+^**, MCV**^−^**TSV**^−^** and MCV**^+^**TSV**^−^** (Table 3).

In group MCV**^+^**TSV**^+^** and in group MCV**^−^**TSV**^−^** the responses with MCV and TSV antigens were similar (Table 4). Interestingly, in group MCV**^−^**TSV**^+^**, TSV-specific IFN-γ responses with TSV antigen were significantly stronger than with MCV antigen at 1.5µg/mL concentration, and also almost significantly stronger at 0.5 µg/ml concentration, whereas the differences with IL-10 responses were less apparent (Table 3). Finally, in group MCV**^+^**TSV**^−^**, IFN-γ and IL-10 responses were significantly stronger with MCV than with TSV at both antigen concentrations (Table 3). In this group the MCV-specific IFN-γ responses were even stronger at 0.5 µg/ml concentration than the TSV antigen specific responses at 1.5 µg/ml antigen concentration (P = 0.007) (Table 3).

### MCV and TSV Specific IFN-γ and IL-10 Responses in the MCC Patient

Th-cell mediated immune responses were studied in a subject with Merkel cell carcinoma whose clinical details are shown in Note S1. Similarly to the healthy controls in group MCV**^+^**TSV**^−^** described above, also this patient was seropositive for MCV and seronegative for TSV, and much stronger IFN-γ and IL-10 responses were found with MCV than with TSV (Note S1). The kinetics of his MCV-specific IL-10 response was similar to that in healthy controls (5d response higher than 3d response); with TSV-antigen a reverse pattern was detected (Note S1).

## Discussion

Immune responses to viruses associated with cancer may reveal valuable information on the pathogenesis of these diseases. Cellular arm of immunity appears elemental in preventing both TSV and MCV-mediated diseases, as immunosuppression predisposes to diseases associated with these viruses, *trichodysplasia spinulosa* and MCC, respectively. The presence of MCV-specific antibodies does not prevent the development of MCC [10], [46]. However, in a recent study using synthetic peptides as antigens it was shown that MCC tumors ultimately develop despite the presence of intra tumor CD8^+^ cells specific for MCV T-Ag oncoprotein [26]. Several immune evasion mechanisms were proposed to explain this: low expression of MHC I in MCC, defective homing or effector functions of CD8^+^ T cells or induction of regulatory-like CD4^+^ T cells which might promote tumor growth [26]. Respective data are not available on TSV.

The present study is the first to describe TSV-specific cellular immunity. T-cell subset depletion and antibody blocking experiments showed that TSV-specific responses were mediated by CD4^+^ Th-cells. Moreover, the comparisons between TSV- and MCV-specific responses further extend the knowledge about MCV-specific Th- cell immunity. Besides helping B-cell immunity and providing antiviral cytokines, Th-cells are essential for generation of memory CD8**^+^** T-cells [24], [47], [48]. In humans, also the secondary activation of memory CD8**^+^** T cells has shown to be Th-cell dependent, and importantly, Th- and cytotoxic cells can recognize different antigens during this process [49]. Therefore, it appears likely that upon simultaneous presentation of T-cell epitopes from both VP1 and LT -proteins, the VP1 protein-specific Th-cells described here may also provide help for cytotoxic (CD8**^+^**) T-cells recognizing epitopes within the nonstructural (LT) proteins, [49].

Highly purified virus-like particles (VLPs) were used as antigens. Similarly to proteins from infectious viruses, also the VLP-derived proteins need prior processing to antigenic peptides by the antigen presenting cells. This is a major difference to approaches using exogenous synthetic peptides, which bypass this initial step of antigen processing, and therefore, are less natural antigens. However, peptides are optimal tools for T-cell epitope mapping.

TSV-specific Th-cell responses did not differ significantly between virus-seropositive and seronegative subjects, albeit the average TSV-specific Th-cell responses were higher among the TSV seropositives than the seronegatives. The presence of TSV-seronegative responders may not fully explain this finding, as respective MCV-seronegative responders were also detectable with the MCV-antigen. Interestingly, however, the vigour of TSV-specific humoral responses had a significant impact on TSV-specific Th-cell responses, and subjects with highest antibody responses not only had significantly stronger responses than the seronegative controls, but also significantly stronger responses than the TSV-seropositive subjects with lower TSV-specific IgG level. At least two different things could account for this. First, subjects with lower TSV-IgG and Th-cell response level may have contracted the TSV infection long time before the subjects with stronger responses became infected. Consistent with this, antibody and Th-cell responses are known to decline with time [50]. Second and more interesting explanation is that subjects with higher TSV-specific responses could have had more reactivations or even reinfections, each having a potential to boost both humoral and cellular responses [51].

An association between virus specific Th-cell responses and antibody responses was found also with MCV, yet it was less evident than with TSV. In contrast to TSV, MCV-specific cytokine responses were significantly stronger in the MCV-seropositive than seronegative subjects, even when MCV**^+^**TSV**^−^** and TSV**^+^**MCV**^−^** subjects were compared.

Data from subgroup experiments showed that if there is any crossreactivity between TSV and MCV, it must be relatively low, since the MCV-serostatus had a significant impact on TSV-specific responses only when double (TSV**^+^** MCV**^+^**) seropositive subjects were compared with double (TSV**^−^**MCV**^−^**) seronegative subjects. Furthermore, some of these double seronegatives showed significant Th-cell responses to both viruses, despite being seronegative to both. This could imply that B-cell immunity against these antigens would persist a shorter time than T cell immunity, or that VP1 proteins of MCV and TSV would contain Th-cell epitopes crossreactive with some other agents, such as other human polyomaviruses (Table S3). Because a high level of cross-reactivity is an essential feature of the T-cell receptor, it is also possible that the Th-cells of our seronegative subjects had originally been primed by Th-cell epitopes differing largely in sequence from the MCV or TSV VP1 proteins [52]–[55]. The most interesting possibility is that in some subjects MCV and/or TSV infection induces cytotoxic CD4^+^ cells which would kill antigen-presenting B-cells and cause eradication of virus specific antibody response [56], [57]. With the (MCV**^+^**TSV**^−^**) MCC patient an early and exceptionally strong IL-10 response was detected with TSV-antigen, suggesting that T-cell epitopes within TSV were recognized as altered peptide ligands [58] or alternatively, these common epitopes activated regulatory T-cells in this patient [59]. In both cases an IL-10 oriented T-cell response is known to occur [58], [59].

Taken together, Th-cell immunity appears to be much better maintained against the major structural protein (VP1) of MCV than TSV. As TSV and MCV infections appear to occur around same age [21], [22], [43], the time span from the primary infection should not explain this. Possibly MCV becomes reactivated in the human body more readily than TSV, boosting Th-cell immunity more efficiently. Comparison of TSV and MCV responses within the MCV**^+^**TSV**^−^** subjects (including the MCC patient) and within the TSV**^+^**MCV**^−^** subjects clearly showed that VP1 proteins from both viruses contain unique Th-cell epitopes which are not shared. However, as the differences between MCV and TSV responses were more significant in the MCV**^+^**TSV**^−^** than the TSV**^+^**MCV**^−^** group, MCV VP1 appears to contain more unique virus specific epitopes than TSV-VP1 does. Alternatively, Th-cell epitopes within MCV VP1 have higher affinity for MHC II than Th-cell epitopes of TSV VP1.

Finally, as CD8**^+^** cells specific for MCV T-Ag oncoprotein clearly provide an important defence against established MCC [26], the MCV VP1-specific Th-cells may be important in preventing the full process of oncogenesis, by suppressing MCV replication with antiviral cytokines such as IFN-γ. If this mechanism exists, also the MCV-crossreactive Th-cells should have a cross-protective role, and subjects with these cells might be less susceptible to develop MCC.

## Supporting Information

Figure S1 **(A) Effect of MCV-specific IgG level on Th-cell responses and (B) comparison of MCV-specific IFN-γ and IL-10 responses among low and high MCV-IgG and MCV-seronegative groups.**
(PDF)

Table S1 **Comparison of MCV-specific IFN-γ and IL-10 responses among 24 MCV-seropositive and 27 seronegative subjects against 0.5 and 1.5 µg/ml MCV VP1 and** ***Candida albicans*** **(2.5 µg/ml) antigen concentration.**
(PPT)

Table S2 **Comparison of IFN-γ and IL-10 responses against MCV VP1 and** ***Candida albicans*** **among MCV^+^TSV^−^ and MCV ^–^TSV^+^ groups.**
(PPT)

Table S3 **Comparison of VP1 amino acid sequence of MCV and TSV with other polyomaviruses.**
(PPT)

Note S1 **MCV and TSV-specific Th-cell responses and clinical information about MCC patient.**
(PDF)

## References

[1] Feng H, Shuda M, Chang Y, Moore PS (2008) Clonal integration of a polyomavirus in human merkel cell carcinoma. Science 319: 1096–1100. 18202256 10.1126/science.1152586 PMC2740911

[2] Shuda M, Feng H, Kwun HJ, Rosen ST, Gjoerup O, et al (2008) T antigen mutations are a human tumor-specific signature for merkel cell polyomavirus. Proc Natl Acad Sci U S A 105: 16272–16277. 18812503 10.1073/pnas.0806526105 PMC2551627

[3] Foulongne V, Kluger N, Dereure O, Brieu N, Guillot B, et al (2008) Merkel cell polyomavirus and merkel cell carcinoma, france. Emerg Infect Dis 14: 1491–1493. 18760031 10.3201/eid1409.080651 PMC2603124

[4] Becker JC, Houben R, Ugurel S, Trefzer U, Pfohler C, et al (2009) MC polyomavirus is frequently present in merkel cell carcinoma of european patients. J Invest Dermatol 129: 248–250. 18633441 10.1038/jid.2008.198

[5] Garneski KM, Warcola AH, Feng Q, Kiviat NB, Leonard JH, et al (2009) Merkel cell polyomavirus is more frequently present in north american than australian merkel cell carcinoma tumors. J Invest Dermatol 129: 246–248. 18650846 10.1038/jid.2008.229 PMC2605200

[6] Paolini F, Donati P, Amantea A, Bucher S, Migliano E, et al (2011) Merkel cell polyomavirus in merkel cell carcinoma of italian patients. Virol J 8: 103. 21385344 10.1186/1743-422X-8-103 PMC3059286

[7] Jung HS, Choi YL, Choi JS, Roh JH, Pyon JK, et al (2011) Detection of merkel cell polyomavirus in merkel cell carcinomas and small cell carcinomas by PCR and immunohistochemistry. Histol Histopathol 26: 1231–1241. 21870327 10.14670/HH-26.1231

[8] Sihto H, Kukko H, Koljonen V, Sankila R, Bohling T, et al (2009) Clinical factors associated with merkel cell polyomavirus infection in merkel cell carcinoma. J Natl Cancer Inst 101: 938–945. 19535775 10.1093/jnci/djp139

[9] Kean JM, Rao S, Wang M, Garcea RL (2009) Seroepidemiology of human polyomaviruses. PLoS Pathog 5: e1000363. 19325891 10.1371/journal.ppat.1000363 PMC2655709

[10] Tolstov YL, Pastrana DV, Feng H, Becker JC, Jenkins FJ, et al (2009) Human merkel cell polyomavirus infection II. MCV is a common human infection that can be detected by conformational capsid epitope immunoassays. Int J Cancer 125: 1250–1256. 19499548 10.1002/ijc.24509 PMC2747737

[11] Touze A, Le Bidre E, Laude H, Fleury MJ, Cazal R, et al (2011) High levels of antibodies against merkel cell polyomavirus identify a subset of patients with merkel cell carcinoma with better clinical outcome. J Clin Oncol 29: 1612–1619. 21422439 10.1200/JCO.2010.31.1704

[12] Carter JJ, Paulson KG, Wipf GC, Miranda D, Madeleine MM, et al (2009) Association of merkel cell polyomavirus-specific antibodies with merkel cell carcinoma. J Natl Cancer Inst 101: 1510–1522. 19776382 10.1093/jnci/djp332 PMC2773184

[13] Schowalter RM, Pastrana DV, Pumphrey KA, Moyer AL, Buck CB (2010) Merkel cell polyomavirus and two previously unknown polyomaviruses are chronically shed from human skin. Cell Host Microbe 7: 509–515. 20542254 10.1016/j.chom.2010.05.006 PMC2919322

[14] van der Meijden E, Janssens RW, Lauber C, Bouwes Bavinck JN, Gorbalenya AE, et al (2010) Discovery of a new human polyomavirus associated with trichodysplasia spinulosa in an immunocompromized patient. PLoS Pathog 6: e1001024. 20686659 10.1371/journal.ppat.1001024 PMC2912394

[15] Haycox CL, Kim S, Fleckman P, Smith LT, Piepkorn M, et al (1999) Trichodysplasia spinulosa–a newly described folliculocentric viral infection in an immunocompromised host. J Investig Dermatol Symp Proc 4: 268–271. 10.1038/sj.jidsp.5640227 10674379

[16] Wyatt AJ, Sachs DL, Shia J, Delgado R, Busam KJ (2005) Virus-associated trichodysplasia spinulosa. Am J Surg Pathol 29: 241–246. 15644782 10.1097/01.pas.0000149691.83086.dc

[17] Tan BH, Busam KJ (2011) Virus-associated trichodysplasia spinulosa. Adv Anat Pathol 18(6): 450–453. 21993271 10.1097/PAP.0b013e318234aad2

[18] Matthews MR, Wang RC, Reddick RL, Saldivar VA, Browning JC (2011) Viral-associated trichodysplasia spinulosa: A case with electron microscopic and molecular detection of the trichodysplasia spinulosa-associated human polyomavirus. J Cutan Pathol 38: 420–431. 21251037 10.1111/j.1600-0560.2010.01664.x PMC3756806

[19] Moens U, Ludvigsen M, Van Ghelue M (2011) Human polyomaviruses in skin diseases. Patholog Res Int 2011: 123491. 21941687 10.4061/2011/123491 PMC3173887

[20] Kazem S, van der Meijden E, Kooijman S, Rosenberg AS, Hughey LC, et al (2012) Trichodysplasia spinulosa is characterized by active polyomavirus infection. J Clin Virol 53: 225–230. 22196870 10.1016/j.jcv.2011.11.007

[21] van der Meijden E, Kazem S, Burgers MM, Janssens R, Bouwes Bavinck JN, et al (2011) Seroprevalence of trichodysplasia spinulosa-associated polyomavirus. Emerg Infect Dis 17: 1355–1363. 21801610 10.3201/eid1708.110114 PMC3381547

[22] Chen T, Mattila PS, Jartti T, Ruuskanen O, Soderlund-Venermo M, et al (2011) Seroepidemiology of the newly found trichodysplasia spinulosa-associated polyomavirus. J Infect Dis 204: 1523–1526. 21926381 10.1093/infdis/jir614

[23] Zhu J, Paul WE (2008) CD4 T cells: Fates, functions, and faults. Blood 112: 1557–1569. 18725574 10.1182/blood-2008-05-078154 PMC2518872

[24] Swain SL, McKinstry KK, Strutt TM (2012) Expanding roles for CD4(+) T cells in immunity to viruses. Nat Rev Immunol 12: 136–148. 22266691 10.1038/nri3152 PMC3764486

[25] Kumar A, Chen T, Pakkanen S, Kantele A, Soderlund-Venermo M, et al (2011) T-helper cell-mediated proliferation and cytokine responses against recombinant merkel cell polyomavirus-like particles. PLoS One 6: e25751. 21991346 10.1371/journal.pone.0025751 PMC3185038

[26] Iyer JG, Afanasiev OK, McClurkan C, Paulson K, Nagase K, et al (2011) Merkel cell polyomavirus-specific CD8 and CD4 T-cell responses identified in merkel cell carcinomas and blood. Clin Cancer Res 17: 6671–6680. 21908576 10.1158/1078-0432.CCR-11-1513 PMC3207011

[27] Tannenbaum CS, Hamilton TA (2000) Immune-inflammatory mechanisms in IFNgamma-mediated anti-tumor activity. Semin Cancer Biol 10: 113–123. 10936062 10.1006/scbi.2000.0314

[28] Boehm U, Klamp T, Groot M, Howard JC (1997) Cellular responses to interferon-gamma. Annu Rev Immunol 15: 749–795. 9143706 10.1146/annurev.immunol.15.1.749

[29] Kaplan DH, Shankaran V, Dighe AS, Stockert E, Aguet M, et al (1998) Demonstration of an interferon gamma-dependent tumor surveillance system in immunocompetent mice. Proc Natl Acad Sci U S A 95: 7556–7561. 9636188 10.1073/pnas.95.13.7556 PMC22681

[30] Kornacker M, Moldenhauer G, Herbst M, Weilguni E, Tita-Nwa F, et al (2006) Cytokine-induced killer cells against autologous CLL: Direct cytotoxic effects and induction of immune accessory molecules by interferon-gamma. Int J Cancer 119: 1377–1382. 16642465 10.1002/ijc.21994

[31] Beatty GL, Paterson Y (2001) Regulation of tumor growth by IFN-gamma in cancer immunotherapy. Immunol Res 24: 201–210. 11594457 10.1385/IR:24:2:201

[32] Willmes C, Adam C, Alb M, Volkert L, Houben R, et al (2012) Type I and II IFNs inhibit merkel cell carcinoma via modulation of the merkel cell polyomavirus T antigens. Cancer Res 72: 2120–2128. 22389452 10.1158/0008-5472.CAN-11-2651

[33] Couper KN, Blount DG, Riley EM (2008) IL-10: The master regulator of immunity to infection. J Immunol 180: 5771–5777. 18424693 10.4049/jimmunol.180.9.5771

[34] Mosser DM, Zhang X (2008) Interleukin-10: New perspectives on an old cytokine. Immunol Rev 226: 205–218. 19161426 10.1111/j.1600-065X.2008.00706.x PMC2724982

[35] Rousset F, Garcia E, Defrance T, Peronne C, Vezzio N, et al (1992) Interleukin 10 is a potent growth and differentiation factor for activated human B lymphocytes. Proc Natl Acad Sci U S A 89: 1890–1893. 1371884 10.1073/pnas.89.5.1890 PMC48559

[36] McKenzie AN, Culpepper JA, de Waal Malefyt R, Briere F, Punnonen J, et al (1993) Interleukin 13, a T-cell-derived cytokine that regulates human monocyte and B-cell function. Proc Natl Acad Sci U S A 90: 3735–3739. 8097324 10.1073/pnas.90.8.3735 PMC46376

[37] Wynn TA (2003) IL-13 effector functions. Annu Rev Immunol 21: 425–456. 12615888 10.1146/annurev.immunol.21.120601.141142

[38] Punnonen J, Aversa G, Cocks BG, McKenzie AN, Menon S, et al (1993) Interleukin 13 induces interleukin 4-independent IgG4 and IgE synthesis and CD23 expression by human B cells. Proc Natl Acad Sci U S A 90: 3730–3734. 8097323 10.1073/pnas.90.8.3730 PMC46375

[39] Kapp U, Yeh WC, Patterson B, Elia AJ, Kagi D, et al (1999) Interleukin 13 is secreted by and stimulates the growth of hodgkin and reed-sternberg cells. J Exp Med 189: 1939–1946. 10377189 10.1084/jem.189.12.1939 PMC2192965

[40] Terabe M, Park JM, Berzofsky JA (2004) Role of IL-13 in regulation of anti-tumor immunity and tumor growth. Cancer Immunol Immunother 53: 79–85. 14610620 10.1007/s00262-003-0445-0 PMC11034335

[41] Ma HL, Whitters MJ, Jacobson BA, Donaldson DD, Collins M, et al (2004) Tumor cells secreting IL-13 but not IL-13Ralpha2 fusion protein have reduced tumorigenicity in vivo. Int Immunol 16: 1009–1017. 15184346 10.1093/intimm/dxh105

[42] Sadeghi M, Riipinen A, Vaisanen E, Chen T, Kantola K, et al (2010) Newly discovered KI, WU, and merkel cell polyomaviruses: No evidence of mother-to-fetus transmission. Virol J 7: 251. 20860804 10.1186/1743-422X-7-251 PMC2955715

[43] Chen T, Hedman L, Mattila PS, Jartti T, Ruuskanen O, et al (2011) Serological evidence of merkel cell polyomavirus primary infections in childhood. J Clin Virol 50: 125–129. 21094082 10.1016/j.jcv.2010.10.015

[44] Franssila R, Hokynar K, Hedman K (2001) T helper cell-mediated in vitro responses of recently and remotely infected subjects to a candidate recombinant vaccine for human parvovirus b19. J Infect Dis 183: 805–809. 11181158 10.1086/318819

[45] Kumar A, Filippone C, Lahtinen A, Hedman L, Soderlund-Venermo M, et al (2011) Comparison of th-cell immunity against human bocavirus and parvovirus B19: Proliferation and cytokine responses are similar in magnitude but more closely interrelated with human bocavirus. Scand J Immunol 73: 135–140. 21198754 10.1111/j.1365-3083.2010.02483.x

[46] Pastrana DV, Tolstov YL, Becker JC, Moore PS, Chang Y, et al (2009) Quantitation of human seroresponsiveness to merkel cell polyomavirus. PLoS Pathog 5: e1000578. 19750217 10.1371/journal.ppat.1000578 PMC2734180

[47] Bennett SR, Carbone FR, Karamalis F, Miller JF, Heath WR (1997) Induction of a CD8+ cytotoxic T lymphocyte response by cross-priming requires cognate CD4+ T cell help. J Exp Med 186: 65–70. 9206998 10.1084/jem.186.1.65 PMC2198964

[48] Janssen EM, Lemmens EE, Wolfe T, Christen U, von Herrath MG, et al (2003) CD4+ T cells are required for secondary expansion and memory in CD8+ T lymphocytes. Nature 421: 852–856. 12594515 10.1038/nature01441

[49] Salkowitz JR, Sieg SF, Harding CV, Lederman MM (2004) In vitro human memory CD8 T cell expansion in response to cytomegalovirus requires CD4+ T cell help. J Infect Dis 189: 971–983. 14999599 10.1086/382032

[50] Sester M, Sester U, Alarcon Salvador S, Heine G, Lipfert S, et al (2002) Age-related decrease in adenovirus-specific T cell responses. J Infect Dis 185: 1379–1387. 11992271 10.1086/340502

[51] Jackson SE, Mason GM, Wills MR (2011) Human cytomegalovirus immunity and immune evasion. Virus Res 157: 151–160. 21056604 10.1016/j.virusres.2010.10.031

[52] Wucherpfennig KW, Strominger JL (1995) Molecular mimicry in T cell-mediated autoimmunity: Viral peptides activate human T cell clones specific for myelin basic protein. Cell 80: 695–705. 7534214 10.1016/0092-8674(95)90348-8 PMC7133435

[53] Hemmer B, Vergelli M, Gran B, Ling N, Conlon P, et al (1998) Predictable TCR antigen recognition based on peptide scans leads to the identification of agonist ligands with no sequence homology. J Immunol 160: 3631–3636. 9558061

[54] Lang HL, Jacobsen H, Ikemizu S, Andersson C, Harlos K, et al (2002) A functional and structural basis for TCR cross-reactivity in multiple sclerosis. Nat Immunol 3: 940–943. 12244309 10.1038/ni835

[55] Mycko MP, Waldner H, Anderson DE, Bourcier KD, Wucherpfennig KW, et al (2004) Cross-reactive TCR responses to self antigens presented by different MHC class II molecules. J Immunol 173: 1689–1698. 15265898 10.4049/jimmunol.173.3.1689

[56] Zhao DM, Thornton AM, DiPaolo RJ, Shevach EM (2006) Activated CD4+CD25+ T cells selectively kill B lymphocytes. Blood 107: 3925–32. 16418326 10.1182/blood-2005-11-4502 PMC1895290

[57] Nikiforow S, Bottomly K, Miller G (2001) CD4+ T-cell effectors inhibit Epstein-Barr virus-induced B-cell proliferation. J Virol 75: 3740–52. 11264363 10.1128/JVI.75.8.3740-3752.2001 PMC114865

[58] Sloan-Lancaster J, Allen PM (1996) Altered peptide ligand-induced partial T cell activation: Molecular mechanisms and role in T cell biology. Annu Rev Immunol 14: 1–27. 8717505 10.1146/annurev.immunol.14.1.1

[59] MacDonald AJ, Duffy M, Brady MT, McKiernan S, Hall W, et al (2002) CD4 T helper type 1 and regulatory T cells induced against the same epitopes on the core protein in hepatitis C virus-infected persons. J Infect Dis 185: 720–727. 11920289 10.1086/339340

