# Supplementary material for: Trichodysplasia spinulosa-Associated Polyomavirus (TSV) and Merkel Cell Polyomavirus: Correlation between Humoral and Cellular Immunity Stronger with TSV
Source: PLoS One. 2012 Sep 24;7(9):e45773. doi: 10.1371/journal.pone.0045773 (PMC3454342; doi:10.1371/journal.pone.0045773)
Supplement: Figure S1 — (A) Effect of MCV-specific IgG level on Th-cell responses and (B) comparison of MCV-specific IFN-γ and IL-10 responses among low and high MCV-IgG and MCV-seronegative groups. (PDF) [file pone.0045773.s001.pdf]

Supplement Figure S1

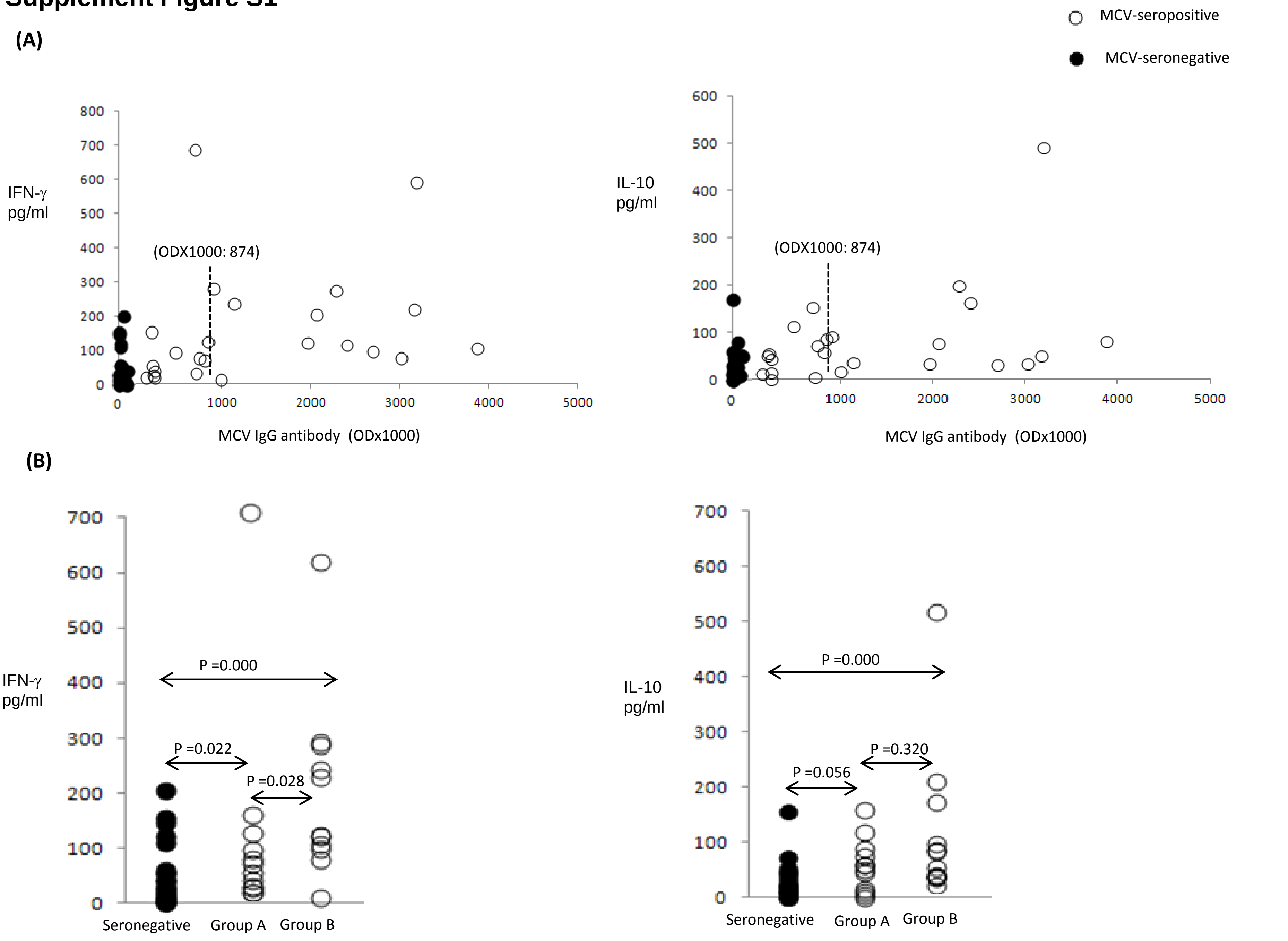

A) **Effect of MCV-specific IgG level on Th-cell responses** . MCV-specific IFN-γ and IL-10 responses were plotted against MCV-IgG responses among seropositive (open circles) and seronegative individuals (closed circles).MCV VP1 specific humoral immune response is shown by optical density x 1000 (ODX1000). Median ODX1000 (874) is shown by (---) a line.

B) Comparison of MCV-specific IFN-γ and IL-10 responses among low and high MCV-IgG and MCV-seronegative groups. MCV-specific IFN-γ and IL-10 responses were compared between the MCV- seronegative subjects and sub-groups of MCV-seropositive subjects. Group A: MCV-seropositive subjects having MCV-IgG ODx1000 below 874, and group B: subjects having MCV-IgG ODX1000 above 874.
