# Supplementary material for: Trichodysplasia spinulosa-Associated Polyomavirus (TSV) and Merkel Cell Polyomavirus: Correlation between Humoral and Cellular Immunity Stronger with TSV
Source: PLoS One. 2012 Sep 24;7(9):e45773. doi: 10.1371/journal.pone.0045773 (PMC3454342; doi:10.1371/journal.pone.0045773)
Supplement: Table S1 — Comparison of MCV-specific IFN-γ and IL-10 responses among 24 MCV-seropositive and 27 seronegative subjects against 0.5 and 1.5 µg/ml MCV VP1 and Candida albicans (2.5 µg/ml) antigen concentration. (PPT) [file pone.0045773.s002.ppt]

## Slide 1
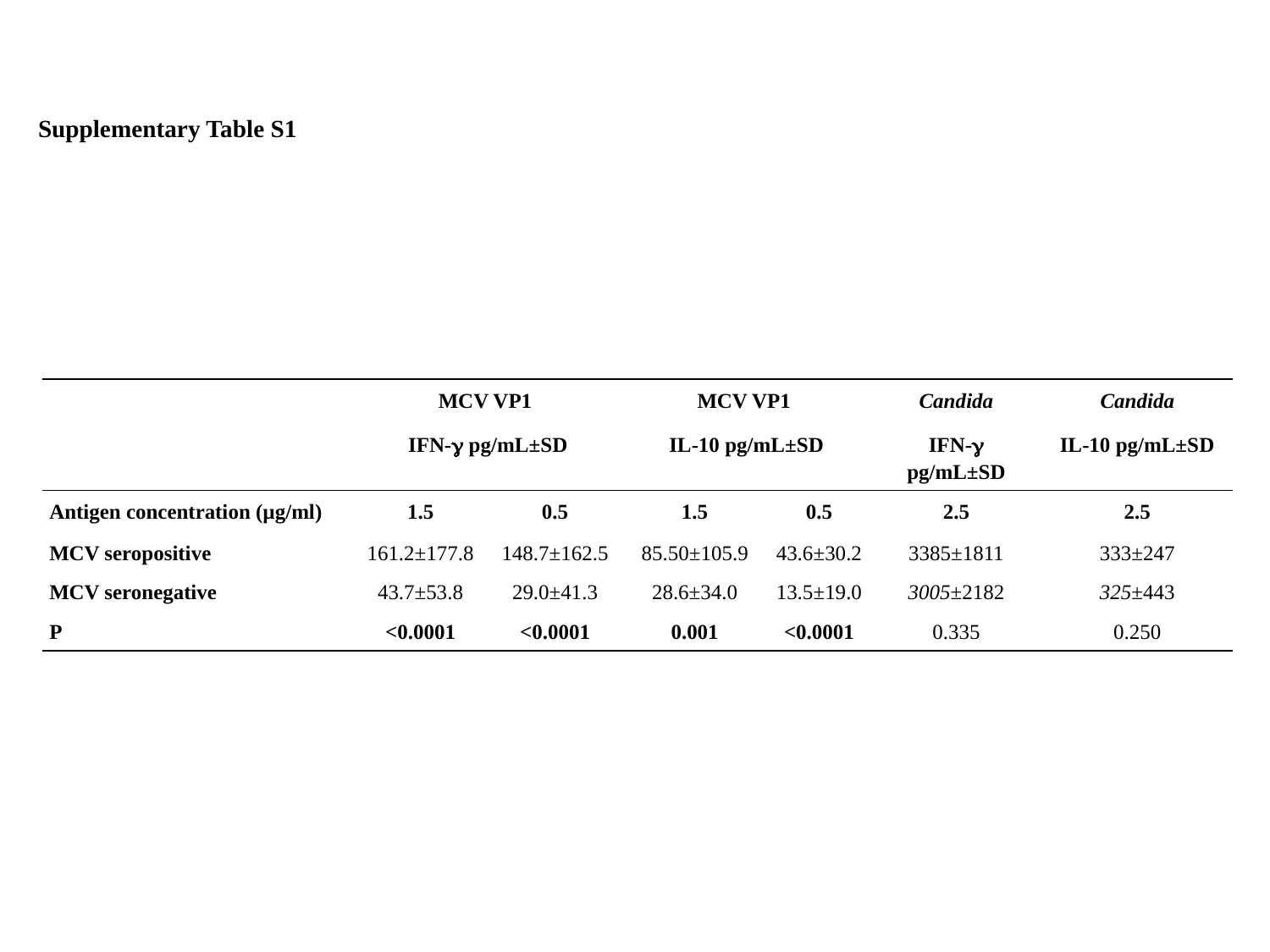

Supplementary Table S1
| | MCV VP1 IFN-γ pg/mL±SD | | MCV VP1 IL-10 pg/mL±SD | | Candida IFN-γ pg/mL±SD | Candida IL-10 pg/mL±SD |
| --- | --- | --- | --- | --- | --- | --- |
| Antigen concentration (µg/ml) | 1.5 | 0.5 | 1.5 | 0.5 | 2.5 | 2.5 |
| MCV seropositive | 161.2±177.8 | 148.7±162.5 | 85.50±105.9 | 43.6±30.2 | 3385±1811 | 333±247 |
| MCV seronegative | 43.7±53.8 | 29.0±41.3 | 28.6±34.0 | 13.5±19.0 | 3005±2182 | 325±443 |
| P | <0.0001 | <0.0001 | 0.001 | <0.0001 | 0.335 | 0.250 |
