# Supplementary material for: Trichodysplasia spinulosa-Associated Polyomavirus (TSV) and Merkel Cell Polyomavirus: Correlation between Humoral and Cellular Immunity Stronger with TSV
Source: PLoS One. 2012 Sep 24;7(9):e45773. doi: 10.1371/journal.pone.0045773 (PMC3454342; doi:10.1371/journal.pone.0045773)
Supplement: Table S2 — Comparison of IFN-γ and IL-10 responses against MCV VP1 and Candida albicans among MCV+TSV− and MCV –TSV+ groups. (PPT) [file pone.0045773.s003.ppt]

## Slide 1
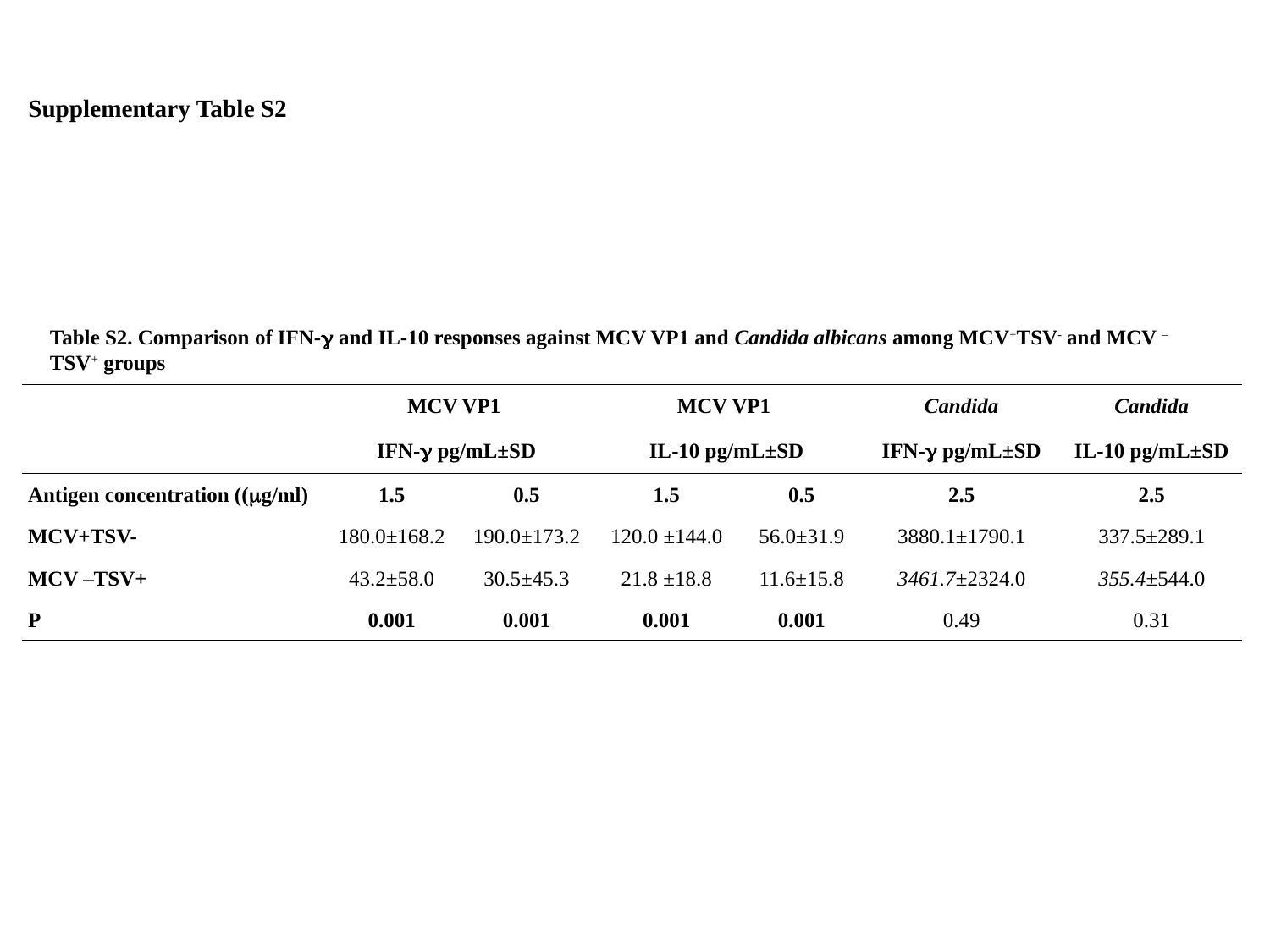

Supplementary Table S2
Table S2. Comparison of IFN-γ and IL-10 responses against MCV VP1 and Candida albicans among MCV+TSV- and MCV –TSV+ groups
| | MCV VP1 IFN-γ pg/mL±SD | | MCV VP1 IL-10 pg/mL±SD | | Candida IFN-γ pg/mL±SD | Candida IL-10 pg/mL±SD |
| --- | --- | --- | --- | --- | --- | --- |
| Antigen concentration ((μg/ml) | 1.5 | 0.5 | 1.5 | 0.5 | 2.5 | 2.5 |
| MCV+TSV- | 180.0±168.2 | 190.0±173.2 | 120.0 ±144.0 | 56.0±31.9 | 3880.1±1790.1 | 337.5±289.1 |
| MCV –TSV+ | 43.2±58.0 | 30.5±45.3 | 21.8 ±18.8 | 11.6±15.8 | 3461.7±2324.0 | 355.4±544.0 |
| P | 0.001 | 0.001 | 0.001 | 0.001 | 0.49 | 0.31 |
