# Supplementary material for: Trichodysplasia spinulosa-Associated Polyomavirus (TSV) and Merkel Cell Polyomavirus: Correlation between Humoral and Cellular Immunity Stronger with TSV
Source: PLoS One. 2012 Sep 24;7(9):e45773. doi: 10.1371/journal.pone.0045773 (PMC3454342; doi:10.1371/journal.pone.0045773)
Supplement: Table S3 — Comparison of VP1 amino acid sequence of MCV and TSV with other polyomaviruses. (PPT) [file pone.0045773.s004.ppt]

## Slide 1
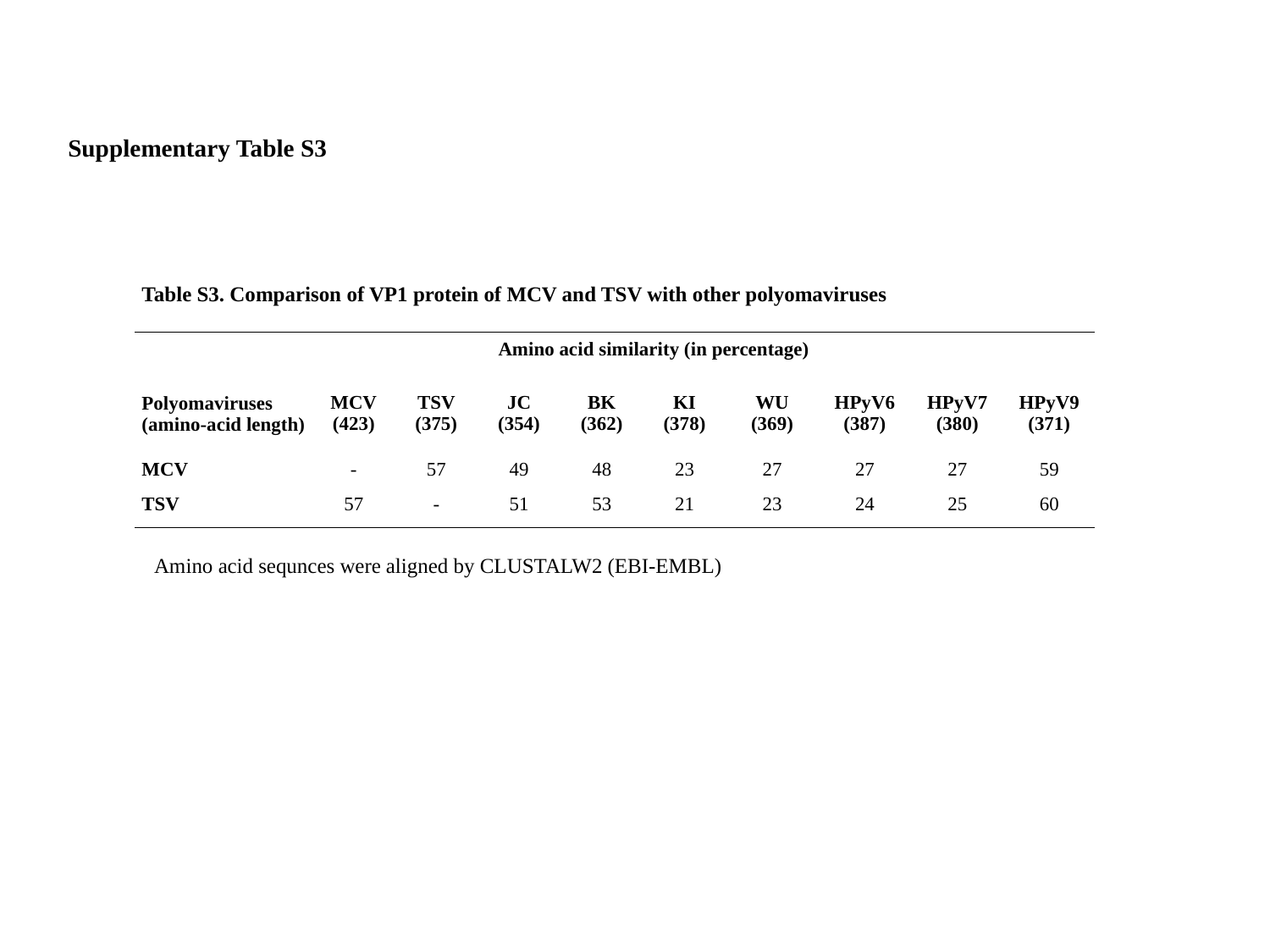

Supplementary Table S3
| Table S3. Comparison of VP1 protein of MCV and TSV with other polyomaviruses | | | | | | | | | |
| --- | --- | --- | --- | --- | --- | --- | --- | --- | --- |
| Amino acid similarity (in percentage) | | | | | | | | | |
| Polyomaviruses (amino-acid length) | MCV (423) | TSV (375) | JC (354) | BK (362) | KI (378) | WU (369) | HPyV6 (387) | HPyV7 (380) | HPyV9 (371) |
| MCV | - | 57 | 49 | 48 | 23 | 27 | 27 | 27 | 59 |
| TSV | 57 | - | 51 | 53 | 21 | 23 | 24 | 25 | 60 |
Amino acid sequnces were aligned by CLUSTALW2 (EBI-EMBL)
