# Supplementary material for: Trichodysplasia spinulosa-Associated Polyomavirus (TSV) and Merkel Cell Polyomavirus: Correlation between Humoral and Cellular Immunity Stronger with TSV
Source: PLoS One. 2012 Sep 24;7(9):e45773. doi: 10.1371/journal.pone.0045773 (PMC3454342; doi:10.1371/journal.pone.0045773)
Supplement: Note S1 — MCV and TSV-specific Th-cell responses and clinical information about MCC patient. (PDF) [file pone.0045773.s005.pdf]

Supplementary Note S1

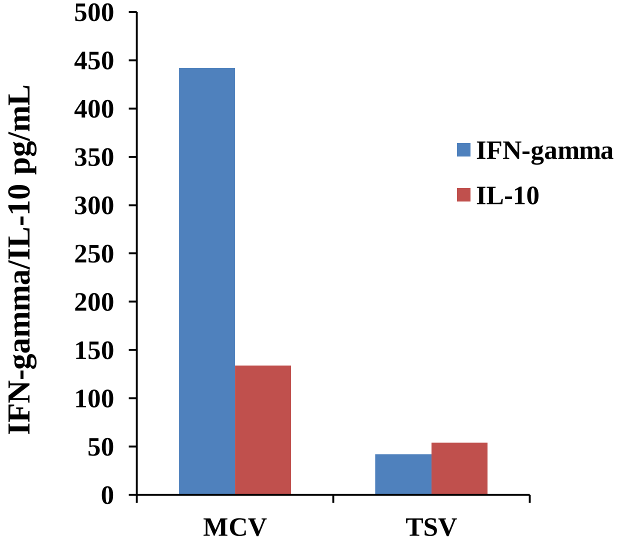

Table: Kinetics of MCV and TSV-specific IL-10 responses in the MCC patient and in healthy control subjects from 3 and 5 day supernatant

		MCV		TSV	
		IL-10 pg/ml		IL-10 pg/ml	
**Serostatus**	Subject	3d	5d	3d	5d
[a]MCV^{+}TSV^{-}	**#T1**	**62**	**134**	**154**	**54**
**MCV^{+}TSV-**	#T43	32	202	12	28
**MCV^{+}TSV^{-}**	#T17	28	60	10	20
**MCV^{+}TSV^{+}**	#T46	10	18	4	30
**MCV^{+}TSV^{+}**	#T16	54	160	28	58
**MCV^{-}TSV^{-}**	#T44	16	24	8	46

a: MCC patient

Fig. MCV and TSV-sepcific Th-cell responses in a MCC tumor patient

Clinical information of MCC patient

Age ,sex and location of tumor: 48 years, male, trunk
Stage of MCC: T3N1aM0; tumor >5cm with nodal metastasis detected in sentinel node biopsy (AJCC staging system) [1]
MCV and TSV serostatus: Pos (MCV), Neg (TSV)
MCV and TSV DNA status: In Blood- MCV (Neg), TSV (Neg)
In Tumor- MCV (Pos), TSV (Neg)
Treatments : Radical surgery with 2 cm marginal, sentinel node biopsy followed by complete lymph node dissection, and post operative radiotherapy
Other information: Patient is alive, follow up time one year.
Immunostaining and histology
Histology consistent with MCC confirmed by immunohistochemistry:
Pansytokeratin +
CK20 +
BCLA2 +
SYP (synpatophysin) +
ChA (chromogranin A) neg
TTF-1 (thyroid transcriptase factor 1) neg

References

1. Allen PJ, Bowne WB, Jaques DP, Brennan MF, Busam K, et al. (2005) Merkel cell carcinoma: Prognosis and treatment of patients from a single institution. J Clin Oncol 23(10): 2300-2309. 10.1200/JCO.2005.02.329.
